# Ectopic pancreas tissue in the posterior mediastinum

**DOI:** 10.1186/s13019-024-02698-8

**Published:** 2024-04-06

**Authors:** Zeynep Berkarda, Jasmina Kuvendjiska, Fabian Bamberg, Elmar Kotter

**Affiliations:** 1 Department of Diagnostic and Interventional Radiology, University Medical Center Freiburg, Freiburg, Germany; 2 Department of General and Visceral Surgery, University Medical Center Freiburg, Freiburg, Germany

**Keywords:** Ectopic pancreas, Computed tomography, Magnetic resonance imaging, Posterior mediastinum, Mediastinitis

## Abstract

The occurrence of ectopic pancreas in the mediastinum is rare. Herein, we report a 22-year-old female who presented with right shoulder pain, dysphagia, fever and headaches. Chest computer tomography revealed a mass in the posterior mediastinum with accompanying signs of acute mediastinitis. Needle biopsy and fine-needle aspiration revealed ectopic gastral tissue and ectopic pancreas tissue, respectively. Surgical resection was attempted due to recurring acute pancreatitis episodes. However, due to chronic-inflammatory adhesions of the mass to the tracheal wall, en-bloc resection was not possible without major tracheal resection. Since then, recurring pancreatitis episodes have been treated conservatively with antibiotics. We report this case due to its differing clinical and radiological findings in comparison to previous case reports, none of which pertained a case of ectopic pancreas tissue in the posterior mediastinum with recurring acute pancreatitis and mediastinitis.

## Introduction

Ectopic pancreas (EP) is defined as pancreatic tissue located outside the normal anatomical location, which has no relevant structural connections to the pancreas. It is most commonly reported in the gastrointestinal tract and is noted in about 2% of autopsies [1]. However, the occurrence of EP in the mediastinum is exceedingly rare, and thus far primarily reported in the anterior mediastinum. In this paper, we present a case of EP located in the posterior mediastinum with recurring acute pancreatitis and mediastinitis, which showed differing radiological and surgical findings and clinical presentation to those in previous case reports.

## Case

**Fig. 1:**
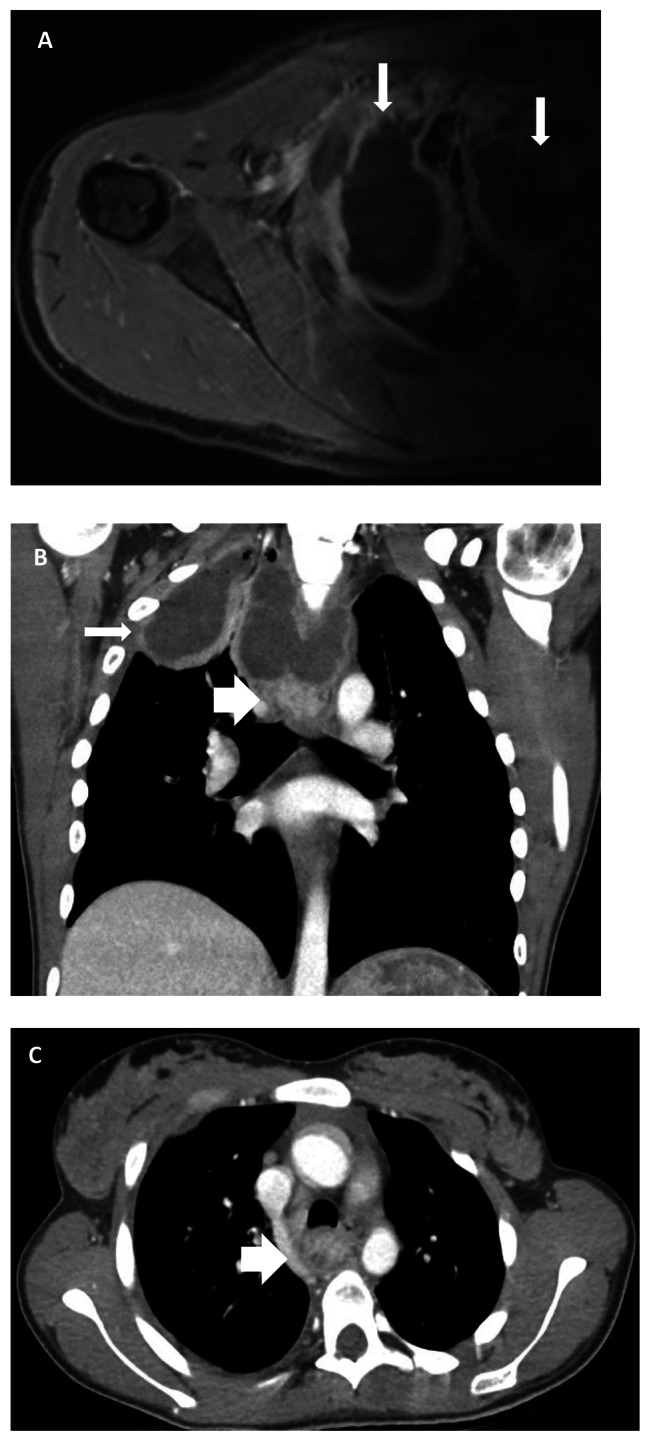
(**A**) MRI indicating a partially captured, ring enhancing abscess formation in the right hemithorax (narrow arrow). (**B**?**C**) CT showing a heterogenously contrasted paratracheal mass in the posterior mediastinum inferiorly adjacent to the abscess formation (broad arrows)

A 22-year-old female was admitted to the referral hospital with acute onset dysphagia, fever, right shoulder pain and headaches. Ultrasound showed small pericardial and bilateral pleural effusions. The white blood cell count (WBC) and C-reactive protein (CRP) were elevated to 19,48 K/μl and 203 mg/l, respectively. Rheumatoid factors were unremarkable. Shoulder magnetic resonance imaging (MRI) with contrast of the right shoulder revealed a partially captured, ring enhancing abscess formation in the right hemithorax and mediastinum. Neck and chest computed tomography (CT) with contrast showed an extensive cervical and mediastinal abscess formation and pleural empyema with multiple air pockets. Notably, an 18 × 20 × 22 mm paratracheal and paraoesophageal mass with heterogeneous contrast enhancement and multiple small, non-enhancing lesions was noted in the posterior mediastinum inferior to the abscess formation (Fig. 1A-C). Collar mediastinotomy was performed for abscess eradication on the same day. The chest was approached by right thoracoscopy two days later. The surgery time was 224 min and blood loss was 150 ml. E. coli was found in the fluid collected intraoperatively, and inflammatory markers reduced markedly after targeted antibiotic treatment with cefuroxime. Post-surgery, the patient underwent an esophagogastroduodenoscopy (EGD) and the mediastinal mass 23 cm aboral the mouth level was biopsied, which revealed ectopic gastral tissue. Bronchoscopy showed a blind-closed Bronchus suis ending in the location of the mediastinal mass.

7 months after discharge the patient was re-admitted with similar complaints of right shoulder pain and dysphagia. WBC and CRP were slightly elevated to 13,16 K/μl and 13 mg/l, respectively. CK-MB isozyme and troponin-T levels were unremarkable. Chest CT was unremarkable apart from the previously noted mediastinal mass. EGD was repeated and endosonographically guided transmural fine-needle aspiration (EUS-guided FNA) puncture was performed. In addition to ectopic gastral tissue, EUS-guided FNA revealed EP parenchyma. Following the EGD the patient self-discharged against medical advice. 9 months later the patient was once again re-admitted with a similar constellation of symptoms. She reported having recurrent episodes of shoulder pain and dysphagia coupled with subfebrile fevers over the course of six months, during which the patient self-administered antibiotics, which usually led to short-term pain relief. Chest CT revealed a new mediastinal abscess formation in the medial right hemithorax adjacent to the EP mass. Serum lipase levels were elevated to 104 U/l. Other serum blood laboratory tests were similar to the initial presentation. The abscess formation was treated conservatively with a course of piperacillin/tazobactam with significant reduction of inflammatory parameters. The patient was discharged one week later.

Due to the increasing frequency of acute pancreatitis episodes, an explorative thoracotomy was performed with the aim of en-bloc-resection. Surgery time was 122 min with minimal blood loss. The chest was approached by right posterolateral thoracotomy through the fourth intercostal space. Intraoperatively, a large semisolid mediastinal mass was noted arising from the posterior mediastinum densely adherent to the trachea and the oesophagus cranial of the azygos vein. The vagal nerve was located anterior to the mediastinal mass. The firm adhesions to the tracheal wall by dense fibrous tissue rendered dissection of the mass from the oesophageal wall and en-bloc-resection unviable without extensive tracheal resection and reconstruction. In accordance with the patient’s wishes, benign histopathological findings and the high risk of peri- and postoperative complications associated with tracheal resection and reconstruction, the surgical team decided against a tracheal resection. The postoperative course was uneventful.

**Fig. 2:**
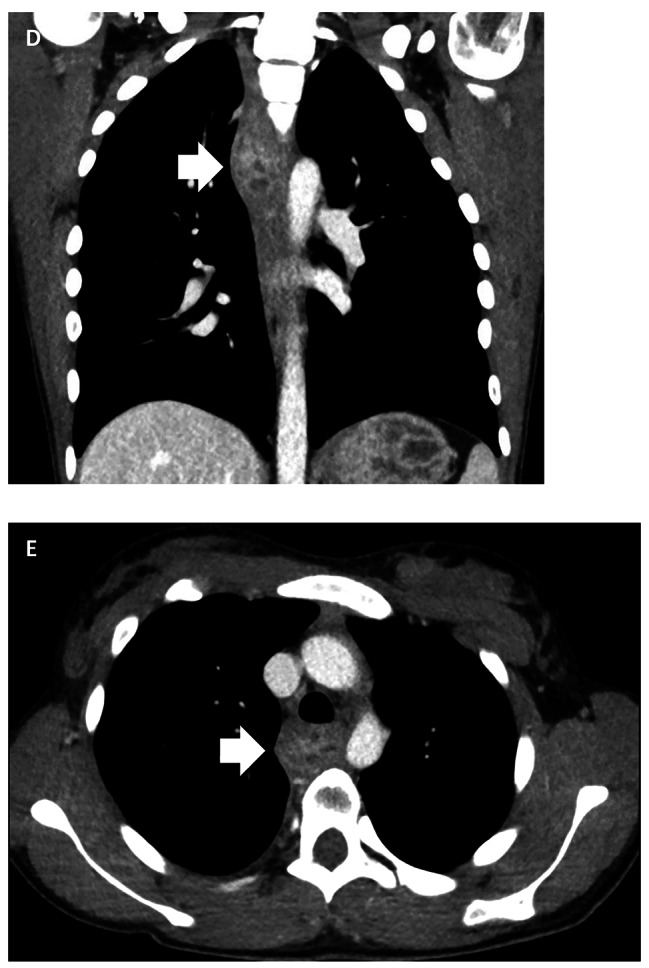
(**D**?**E**) 5-year follow-up computer tomography showing the mediastinal mass with multiple small non-enhancing lesions. The mass demonstrates a minimal growth progression

Following the explorative thoracotomy, the patient has been readmitted thrice to the referral hospital due to acute pancreatitis episodes, each time with a similar clinical presentation to the initial admission, all of which have been treated with targeted antibiotic therapy. Currently, the patient is closely monitored in the pancreas outpatient clinic of the referral hospital. A follow-up chest CT this year showed a minimal growth progress of the mediastinal mass, possibly due to chronic-inflammatory processes (Fig. 2). Biopsies in the follow-up EGDs showed no indication of malignant transformation to date. In the last outpatient appointment, the patient reported that the frequency of the acute pancreatitis episodes had diminished in the past year. The option of resection was re-evaluated and discussed with the patient, who once again decided against a radical surgical resection. A follow-up EGD and magnetic resonance cholangiopancreatography (MRCP) of the abdomen are planned in 2023.

## Discussion

EP is a rare congenital condition, which most commonly occurs in the gastrointestinal tract. EP located in the mediastinum is exceedingly rare and was first described by Shillitoe and Wilson in 1957 [2]. Since Shillitoe and Wilson, a review of the literature revealed over 30 cases of mediastinal EP, with the vast majority of cases reporting EP in the anterior mediastinum, typically in the context of encapsulated cystic lesions or solid-cystic, heterogeneously contrasted masses [3–28], and Rokach et al. have provided a detailed overview of the literature as of 2013 [13]. As of present, the histogenesis is still unclear with two main prevailing theories. The first theory is based on the abnormal differentiation of the ventral primary foregut, the components of which include the pancreas, trachea and oesophagus. The second theory stipulates that pancreatic bud cells migrate to various locations, such as to the mediastinum. In the index patient, the initial needle biopsy revealed ectopic gastric tissue in the mediastinal mass and bronchoscopy showed a blind-closed Bronchus suis ending in the location of the mass. The presence of gastric heterotopia and a congenital bronchial abnormality may favour the former hypothesis of abnormal cell differentiation of the foregut, however, further investigation is necessary to satisfactorily determine the genesis. In the index patient, gastric heterotropia was an incidental finding and complications associated with ectopic gastric tissue such as haemorrhage or haemoptysis have not occurred to date.

Symptoms of mediastinal EP are generally non-specific, including fever, chest pain, dyspnoea and coughing, which may be caused by mechanic compression of the neighbouring structures, such as the trachea. Complaints tend to correspond to the size of the mass and whether infiltrative inflammatory processes are present. Although less common, pericardial and pleural effusions have been also reported, which were also observed in the index patient at initial presentation [17, 29]. Interestingly, in the index patient the leading symptoms were primarily caused by acute-inflammatory processes, and, in contrast to many cases, the index patient did not report dyspnoea, likely because the mass itself was significantly smaller than in most cases. Laboratory findings may be unremarkable. The index patient showed elevated WBC, CRP and serum lipase parameters during recurring pancreatitis episodes. To date, hypoglycaemia as a possible indicator of active insulin producing pancreatic tissue has not been observed.

In contrast to various cases demonstrating a single cystic lesion [4, 6, 8, 9, 12, 14, 15, 21, 26–29, 31], imaging findings of the index patient showed a heterogeneously contrasted paratracheal and –oesophageal mass located the posterior mediastinum with multiple liquefied, non-enhancing lesions, primarily correlating to pseudocysts. The mass did not show a significant compression of the neighboring structures. A similarly irregular, heterogeneously contrasting mass has been reported, likewise with inflammatory adhesions to the neighboring structures, albeit in the anterior mediastinum [7]. An interesting aspect of the CT findings in the index patient are the twice-noted accompanying abscess formations. To the best of our knowledge, there is no reported case of an EP mass in located in the posterior mediastinum with clinical and imaging signs of recurring acute pancreatitis and mediastinitis.

As noted above, acute pancreatitis episodes were treated with antibiotics in the index patient. An antibiotic therapy in acute pancreatitis is only recommended in case of an infection, which is also the standard procedure in our clinical center. In the unique case of our index patient, the pancreatitis episodes were associated with recurring abscess formations and/or mediastinitis and thus merited an antibiotic therapy. Furthermore, since the ectopic pancreas tissue was not resectable without major surgery, other therapeutic options were limited. As a result, the risks of the antibiotic therapy were considered inferior to the risk of mediastinitis or mediastinal abscess in the index patient and pancreatitis episodes have been successfully managed thus far.

The differential diagnosis of posterior mediastinal masses include neurogenic tumors such as schwannoma, neuroblastoma or paraganglioma, non-neurogenic tumors including lymphoma, sarcoma or metastasis, lymphadenopathy and foregut duplication cysts, none of which typically present with CT findings similar to those in the index patient. For example, neurogenic tumors typically present as homogenous attenuated masses, while duplication cysts usually appear as fluid-like sacs with well-defined borders. Furthermore, these entities are not usually associated with acute and chronic inflammatory processes seen in the index patient. However, conclusive diagnosis is dependent on histologic findings, following either surgical resection, core-needle biopsy or EUS-guided FNA. To the best of our knowledge, apart from one case report of aggressive adenocarcinoma of EP in the anterior mediastinum [29], all reported lesions were benign. To date, all follow-up needle biopsies of the index patient have shown benign results. An interesting aspect of our case is that initial needle biopsy solely detected gastric heterotopia, and EP was first diagnosed in the following EUS-guided FNA. To date, only one other case of mediastinal EP has been primarily diagnosed via EUS-guided FNA [30].

Due to the albeit small risk of malignant transformation and to alleviate symptoms, surgical resection of EP is strongly recommended [8, 31]. Another unique aspect of this case is the failure of en-bloc resection without major tracheal resection due to firm fibrous adhesions, most likely caused by chronic-inflammatory processes. In the vast majority of cases, curative en-bloc resection was successfully performed via median sternotomy or thoracotomy with no reported recurrences, apart from the sole case report of aggressive adenocarcinoma which metastasized six months after resection.

## Conclusion

Although exceedingly rare, ectopic pancreatic tissue should be considered in the workup of mediastinal masses, including in the posterior mediastinum. We have presented a rare case of EP in the posterior mediastinum with periodic inflammatory symptoms caused by acute pancreatitis and mediastinitis. Awareness of this rare differential diagnosis and thorough sampling are necessary for correct diagnosis and treatment.

## Data Availability

A copy of the written consent is available for review by the Editor-in-Chief of this journal.
